# A Deep-Learning Based System for Rapid Genus Identification of Pathogens under Hyperspectral Microscopic Images

**DOI:** 10.3390/cells11142237

**Published:** 2022-07-19

**Authors:** Chenglong Tao, Jian Du, Yingxin Tang, Junjie Wang, Ke Dong, Ming Yang, Bingliang Hu, Zhoufeng Zhang

**Affiliations:** 1 Xi’an Institute of Optics and Precision Mechanics, Chinese Academy of Sciences, Xi’an 710119, China; chengltao@126.com (C.T.); dujian@opt.ac.cn (J.D.); gjswjj@gmail.com (J.W.); 2 University of Chinese Academy of Sciences, Beijing 100049, China; 3 Key Laboratory of Biomedical Spectroscopy of Xi’an, Xi’an 710119, China; 4 Independent Researcher, Changsha 410000, China; yxtang1995@163.com; 5 The Second Affiliated Hospital of Air Force Military Medical University, Xi’an 710119, China; drdongke@163.com (K.D.); yangming745@163.com (M.Y.)

**Keywords:** infectious pathogens, hyperspectral microscopy, bacteria identification, artificial intelligence, imaging, genus, spectral characteristics

## Abstract

Infectious diseases have always been a major threat to the survival of humanity. Additionally, they bring an enormous economic burden to society. The conventional methods for bacteria identification are expensive, time-consuming and laborious. Therefore, it is of great importance to automatically rapidly identify pathogenic bacteria in a short time. Here, we constructed an AI-assisted system for automating rapid bacteria genus identification, combining the hyperspectral microscopic technology and a deep-learning-based algorithm Buffer Net. After being trained and validated in the self-built dataset, which consists of 11 genera with over 130,000 hyperspectral images, the accuracy of the algorithm could achieve 94.9%, which outperformed 1D-CNN, 2D-CNN and 3D-ResNet. The AI-assisted system we developed has great potential in assisting clinicians in identifying pathogenic bacteria at the single-cell level with high accuracy in a cheap, rapid and automatic way. Since the AI-assisted system can identify the pathogenic genus rapidly (about 30 s per hyperspectral microscopic image) at the single-cell level, it can shorten the time or even eliminate the demand for cultivating. Additionally, the system is user-friendly for novices.

## 1. Introduction

Infectious diseases are a significant threat to public health and remain the leading cause of death in developing countries, despite remarkable medical progress. Therefore, how to prevent and treat them efficiently is of great significance [1,2,3,4].

Currently, there are several conventional methods for bacteria detection, such as conventional morphological examination [5], polymerase chain reaction (PCR) [6], the matrix assisted Laser Desorption Ionization Time of Flight (MALDI-TOF) [7], biochemical identification [8], serological tests [9], etc. However, a morphological examination is time-consuming, since pathogens must be cultivated to be colonies. PCR, which needs skillful operations, is only adopted for emergencies due to the high expense. Especially, MALDI-TOF is regarded as the “gold standard”. However, it is scarcely used in clinics because of the complicated operation and expensive cost. Additionally, the reproducibility of results is poor due to the destruction to the specimen. As for patients, broad-spectrum antibiotics are first-line-adopted when the cause of infection is unclear. Unfortunately, the treatment seldom brings a satisfactory efficacy. Moreover, it might also bring a heavy financial and psychological burden to the patient and potential risk of worsening the condition [10,11]. Due to the relative scarcity of laboratory physicians, there is an urgent need to rapidly automate pathogenic bacteria identification at a low price.

By utilizing spectral profiles of bacterial suspensions, Dimitris et al. identified five bacterial species [12] and demonstrated that spectral technique could be a rapid, cheap and noninvasive tool for bacterial identification. However, their method has only spectral information and limited recognition capability. To adapt to more complex recognition scenarios, hyperspectral technology (finer spectral resolution than a spectral technique) and imaging technology are combined into hyperspectral imaging (HSI) technology, which was used to identify bacteria with colony hyperspectral images [13,14,15,16]. Since hyperspectral images have three dimensions, similar to a cube, they are also called datacubes.

However, these methods are colony-based and require the culturing of bacteria to a considerable amount, which is very time-consuming. For faster identification of pathogens, HSI and microscopic techniques were combined to develop microscopic hyperspectral imaging (HMI). HMI was exploited to detect food-borne pathogens at the cell level [17,18,19,20]. However, the narrow range of wavelengths (450–800 nm) and coast spectral resolution (4 nm) are insufficient for classifying infectious pathogens. In addition, a microscopic datacube was decomposed or averaged, which destroyed the strong correlations between the spectral and spatial information of the datacube. Although convolutional neural networks (CNNs) were utilized, the small-scale dataset was inadequate for an excellent CNN-based algorithm and clinical practice.

In this study, a new HMI system with a wider wavelength range (440–1023 nm) and finer spectral resolution (2.1 nm) was developed. Over 130,000 HMIs (hyperspectral microscopic images) for bacteria of 11 genera were collected with this equipment. Moreover, a 3D-CNN-based network was proposed, called the Buffer Net, to maintain the data integrity in HMIs. For noninvasive, fast and accurate bacteria detection, the HMI system and trained Buffer Net were integrated to be an AI-assisted system.

In Section 2, we detail the collection and processing of the data, system design and integration. The results re shown in Section 3, and the significance of our work is discussed in Section 4. In Section 5, we concisely conclude the paper.

## 2. Materials and Methods

**Figure 1:**
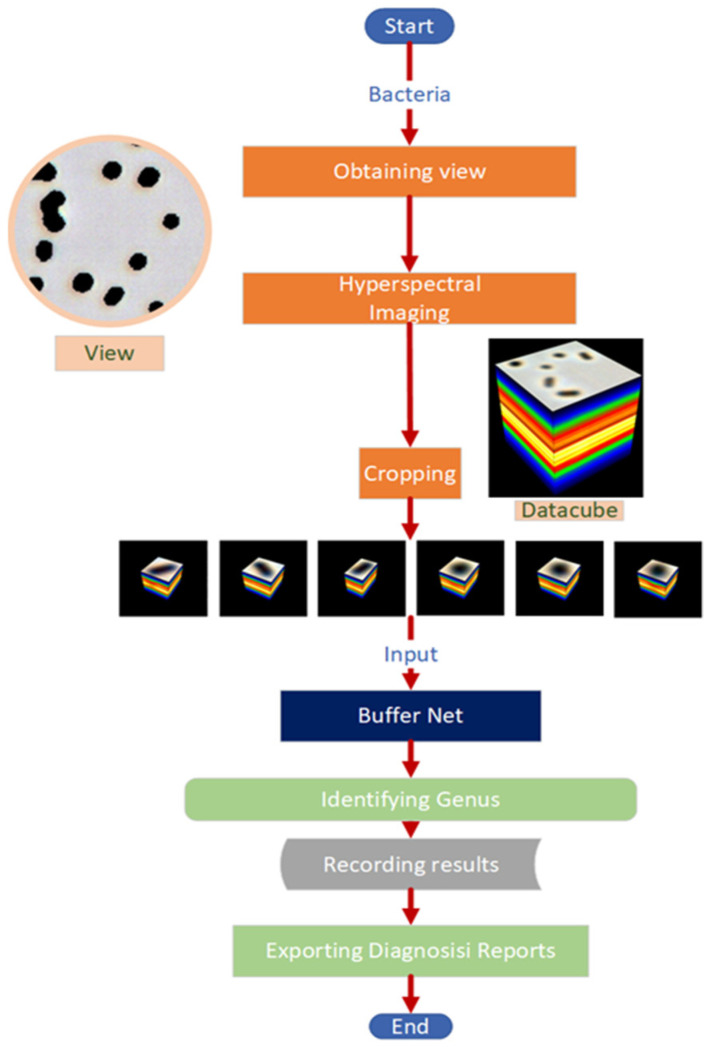
Workflow diagram of the AI-assisted system. The field of view of the bacteria made into slides is acquired under a microscope with 100× magnification and then imaged. The bacteria in the datacube obtained by imaging are cropped out and fed into the Buffer Net. The identification results are recorded and exported to help physicians make a diagnosis.

The workflow diagram of the AI-assisted system is shown in Figure 1. Additionally, visualization of the cropping individual bacteria and the main functions of the system are included.

### 2.1. Bacteria Strains

Most strains in this study were from The Second Affiliated Hospital of Air Force Military Medical University. In addition, in order to increase the diversity of our data, bacteria from the American Type Culture Collection (ATCC) were also adopted in the study. The genera used in this study are summarized in Table 1. The extracted infectious bacteria were purified (if necessary) and incubated on blood plates at a constant temperature of 35–37 °C for 24–48 h. Afterwards, some of the individual colonies were dissolved in turbidity tubes under high pressure with saline (that is, to sterilize). A slide was taken out to have the bacterial suspension in the inoculation loop coated on it evenly. Then, the microscopic slide was dried naturally in the biosafety cabinet. Finally, a 75% alcohol solution was applied to the dried slide, and then, absorbent paper was used to dry the slide. After implementing the above process, the slide was well-prepared for observation and imaging under the HMI system.

### 2.2. Hyperspectral Microscopic Imaging (HMI) System

**Figure 2:**
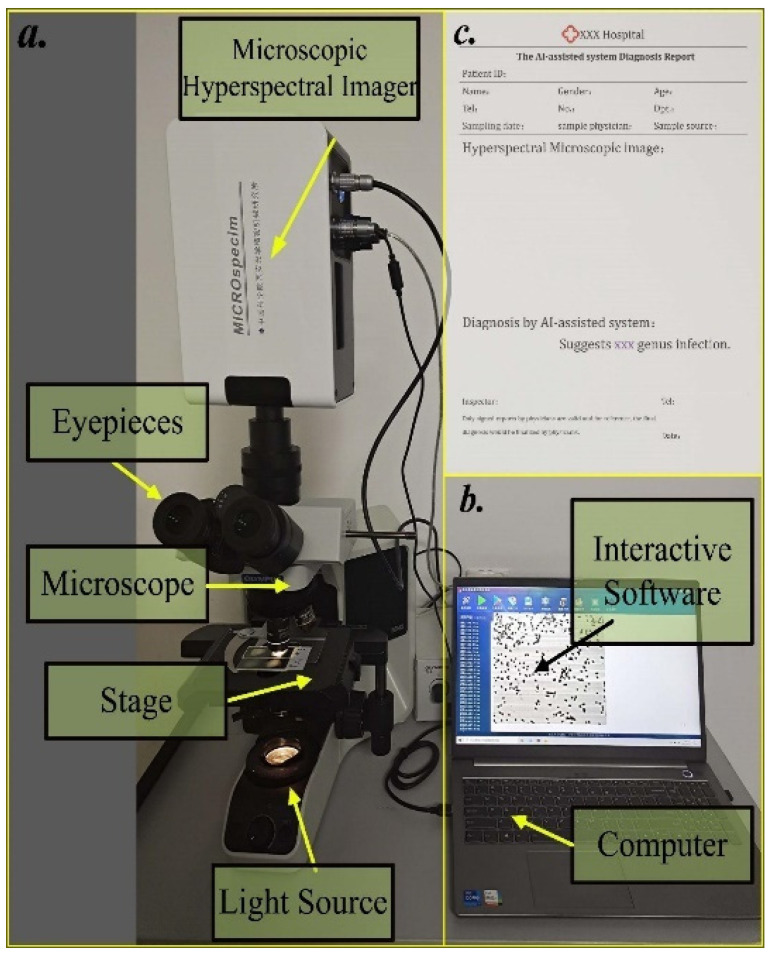
The components of the integrated AI-assisted system. (**a**) The HMI system. It is composed of a microscope and hyperspectral camara and is responsible for detecting and imaging the view containing bacteria. (**b**) The host, where data storage processing, bacteria identification and interaction are deployed. (**c**) A template for the PDF files, i.e., the diagnostic report, output by the AI-assisted system.

A built-in push-broom HMI system (Figure 2a) was developed, which included a microscope and hyperspectral imaging system. An Olympus Biomedical BX43 upright microscope (Olympus, Tokyo, Japan) was used in the system. A 30-W halogen lamp was utilized as the light source. The objective parameters for bright field imaging: 100×/0.90. The model number of the sensor was acA2000-165 umNIR (Basler, Ahrensburg, Germany). The spatial resolution of our hyperspectral camera was about 0.67 µm, the average spectral interval was 2.1 nm, and the field of view (FOV) was 64 × 64 μm^2^. Then, a datacube was obtained containing 277 bands (440–1023 nm).

### 2.3. Data Collection and Preprocessing

Due to the low signal-to-noise ratios of the front and rear bands, the first 50 and the last 27 channels of the 277 bands were discarded. To even out the brightness of the entire image, each scanning column was averaged, and then, the means were divided by each pixel in the scanning column. To avoid the influence of extreme values, the 300 maximum and 300 minimum pixels in the scanning column were excluded when calculating the average. This process is called flat-field correction [21].

Then, a clustering algorithm, K-means [22], was adopted to separate the bacterium from the background in HMIs, and a binary image was obtained. The background was calculated by pixel-wise multiplication on the binary image and the whole datacube. Then, the average background brightness was then calculated.

The image where pixel values were like transmittance was obtained by dividing the average background brightness band-wise. The standardization is in order to eliminate the effects of different lighting. The bacterium was accurately identified by its location and size in the image by finding the connected area in the binary image. Then, the bacteria were selected and cropped from the 200 × 1000 × 1000 datacube (Figure 1). The connected areas at the boundary were discarded due to not being intact. The spectral dimensions of the images obtained for individual bacterium was 200, and there was no fixed value for the length and width of the images due to the different pathogen sizes. Finally, more than 130,000 HMIs of 11 infectious genera were collected and randomly assigned to the training set and test set at 70% and 30%, respectively. The number of samples is presented in Table 1.

### 2.4. Model Design

#### 2.4.1. Deep Learning

Deep learning aims to design a deep network *f* and determine its parameters *θ* and to fit the relationship between the input data *x* and output *y^pre^* as accurately as possible. Deep networks generally consist of three parts: input layer, hidden layers and output layer, all of which are composed of neurons. The input layer is only responsible for the input of data, and the data is propagated in the hidden layer with linear and nonlinear transformations. This is also the process of extracting data features. Finally, the data *x* is mapped as *y^pre^* and output from the output layer. This whole process is the forward propagation of the network. *y^pre^* and *x* correspond to the ground truth *y^t^* to calculate the loss. The loss is input into *f* from the output layer through the hidden layer. This process is the backward propagation of the neural network. During training, after iterating forward and backward propagation, the prediction *y^pre^* for *x* would get closer and closer to the true value *y^t^*. When the condition is satisfied, the training is stopped, and the parameter *θ* is fixed. The trained neural network *f* can be used to predict the *y^test^* value corresponding to the input data *x^test^*.

#### 2.4.2. Buffer Net

As mentioned above, HMIs have three dimensions: spectrum, height and width. The spatial information included in the height and width dimensions is strongly related to the spatial information in the spectral dimension. Extracting the features of HMIs without losing their correlation is extremely important.

Since the 1D-CNN filter is only able to extract the features of one-dimensional spectral profiles and 2D-CNN extracts the spatial features of two-dimensional images, it is not possible for them to extract spectral–spatial features in HMIs [23,24,25]. Therefore, 3D-CNN uses three-dimensional (3D) filters that are suitable to extract the features of datacubes [26]. For a datacube, $D\in R^{S\times H\times W}$, 3D-CNN uses a 3D filter $F\in R^{K\times I\times J}$ to execute the convolution operation. A 3D feature map $M\in R^{U\times V\times Z}$ is obtained. Assuming the stride is *t*, M[s,h,w]  can be computed as Equation (1) when it is a value located at (*s*, *h*, *w*) in *M*. Traverse the image with *F*, and finally, output *M*. Since *D* is not decomposed or averaged, the correlation between the spectral and spatial information is retained.

(1) $$M\left[s,h,w\right]=\left(D*F\right)\left[s\times t,h\times t,w\times t\right]=\overset{K}{\underset{k=1}{\sum }}\overset{I}{\underset{i=1}{\sum }}\overset{J}{\underset{j=1}{\sum }}D\left[s\times t-k,\;h\times t-i,\;w\times t-j\right]\times F\left[k,i,j\right]\;\;\;\;\;\;\;\;\;\;$$

Bacteria in datacubes are small objects with resolutions less than 32 × 32 pixels (defined in [27]), and the resolution of their feature maps will be less than 1 pixel in the feature map after several down samplings. Therefore, two kinds of convolutional layers were designed: a down sampling layer and buffering layer. For expanding the receptive field of the neurons, the stride of the down sampling was set as 2 to down sample the datacube. The stride of the buffering layers is fixed to be 1 for enhancing the representative abilities without reducing the resolution of the feature maps. The convolutional kernels of both layers were 3 × 3 × 3 for extracting detailed information of the datacubes.

**Figure 3:**
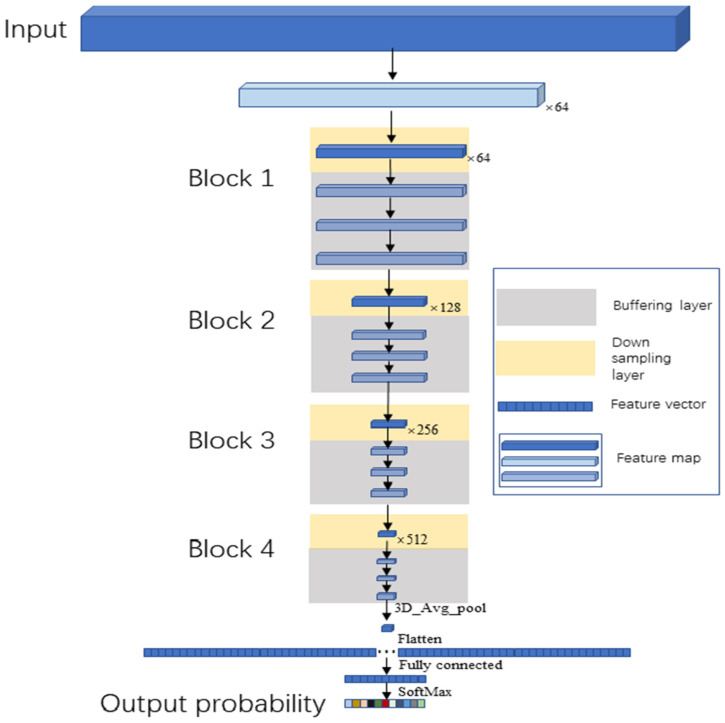
The architecture of the Buffer Net and the data flow. The input was HMI images at a size of 200 × 25 × 25. Firstly, it was down sampled with the output as 64 feature maps, whose sizes were 98 × 25 × 25. Then, they were input to the four blocks to down sample and buffer. The size of the output feature maps for the four blocks were 49 × 13 × 13, 25 × 7 × 7, 13 × 4 × 4 and 7 × 2 × 2, respectively, and the number of output feature maps for the four blocks were 64,128, 256 and 512, respectively. The feature map output by the fourth block was flattened into a vector after a 3D average pooling, which was then input to the fully connected layer. The final output was the probability for each bacterial genus by SoftMax. For accelerating the training process, every 3D convolutional layer was followed by a 3D Batch Normalization layer.

A block was composed of a down sampling layer and three buffering layers. In the Buffer Net (Figure 3), a total of four blocks were included. Moreover, the spectral dimension was compressed when the HMI images came into the network by setting the first filter to down sample images on the spectral dimension with a stride of (2, 1, 1) and a convolutional kernel (5, 1, 1).

The dimensions of the feature maps output by the final block were 7 × 2 × 2. The feature maps were flattened into a vector and input to the SoftMax function through 1536 full connections after a 3D average pooling, which would transfer the 11-way output into eleven probabilities for each bacterial genus, and the final output was the genus with the highest probability. Our network consisted of 17 3D convolutional layers (1 + 4 down sampling layers, 4 × 3 buffering layers), 1 fully connected layer and 1 SoftMax layer.

### 2.5. Development Language and Training Details

In this study, Python was adopted to develop the Buffer Net for identifying bacterial genera. In addition, C# was adopted to develop the interactive software for the AI-assisted system. The Buffer Net was integrated into this software to accomplish the specimen digitization, image analysis and bacteria identification.

The batch size was set to 128. All images were resized to 25 × 25 for accelerating the algorithm training before inputting the model. For training, we set the learning rate to 0.0001, the momentum to 0.9 and the epoch to 30. The optimizer used in the paper is the stochastic gradient descent (SGD). At each epoch, the training samples were reshuffled.

### 2.6. System Integration

The whole bacterial identified system was established by integrating the HMI system, the interactive software with a 3D-CNN-based algorithm (Buffer Net) component, data preprocessing and genus identification. The advantage of the whole system is that it could perform all of these steps in a minute, with 30 s for imaging and another 30 s for data preprocessing and genus identification.

To achieve rapid data storage, a USB 3.0 interface was adopted to connect with the computer, which coincided with the data digitization. Once it was completed, the pseudo color image was automatically produced and shown on the screen. The identification was achieved by clicking on the identification buffer of the user. All information about the cases could be exported by the system in the form of a PDF (Figure 2c). The appearance and components of this system are shown in Figure 2.

Furthermore, to facilitate the subsequent model upgrades, an interface in the software was set up to quickly invoke the model. The relevant model developed by Python, the weight file obtained from training and packaging as a file in the form of .exe were all packed for easy use. The subsequent upgrades would be achieved by replacing the .exe file without much modification to the code.

### 2.7. Evaluation Metrics

In this study, the confusion matrix and overall accuracy were applied to evaluate the performance of the system for differentiating these 11 genera. In the confusion matrix, the ordinate represents the true genus of each category, and the abscissa represents the genus predicted by algorithms. Diagonal elements represent the prediction accuracy of each genus, and nondiagonal elements represent the percentage that the genus on the ordinate was misjudged as the category on the abscissa. The overall accuracy represents the total precision of the system for differentiating all these 11 genera.

The calculation method for the elements in the (*I*, *J*) coordinates in the confusion matrix is as follows:

$$Precision_{ij}=\frac{P_{ij}}{\sum_{k=1}^{11}P_{ik}}\times 100\%,\;\;\left(1\leq i\leq 11,\;1\leq j\leq 11\right).$$

$Precision_{ij}$ represents the percentage where *i* genera were misidentified as *j* genera. *P_ij_* represents the number of genera *i* identified as *j* genera, and *P_ik_* represents the number of genera *i* identified as *k* genera.

In order to evaluate the overall accuracy of the system, we also introduced the overall accuracy, whose calculation formula is as follows:

$$Accuracy=\frac{\sum_{i=1}^{11}P_{ii}}{\sum_{j=1}^{11}\sum_{k=1}^{11}P_{jk}}\times 100\%=\frac{\sum_{i=1}^{11}P_{ii}}{\sum_{j=1}^{11}n_{j}}\times 100\%=\frac{\sum_{i=1}^{11}P_{ii}}{N}\times 100\%$$

*P_ii_* represents the number of genera *i* bacteria correctly classified, and *P_ij_* represents the number of *j* genera predicted as genera *k*. Notably, *j* could be equal to *k*. *n_j_* represents the total number of bacteria diagnosed as *j* genera, and *N* represents the total number of bacteria of all the genera.

## 3. Results

### 3.1. Hyperspectral Microscopic Images

Over 130,000 hyperspectral images of 11 common infectious pathogens were collected with cautiousness (Stenotrophomonas, Escherichia, Morganella, Burkholderia, Serratia, Pseudomonas, Acinetobacter, Klebsiella, Proteus, Staphylococcus and Enterococcus) for individual bacteria through hyperspectral microscopic imaging technology.

**Figure 4:**
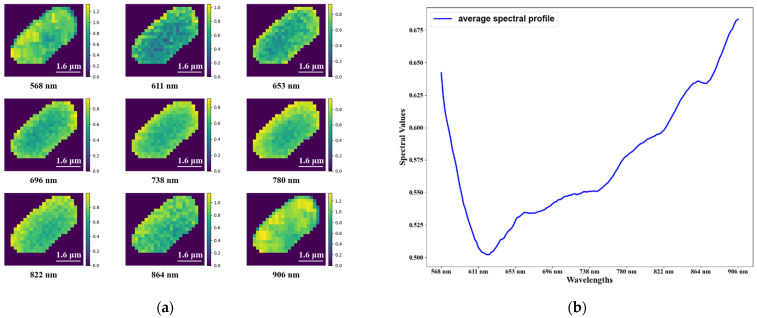
(**a**) The heatmap of a bacterium (Pseudomonas) on several wavelengths. The brightness of the color in the images indicates the intensity of the energy. The background in the heatmap is filled with zero. (**b**) The average spectral profiles for a single bacterium.

The datacube of a bacterium (Pseudomonas) is presented in Figure 4. For ease of observation, several wavelength images of the datacube are presented separately. Moreover, the mean spectrum of a single bacterium is also visualized.

### 3.2. Classification Performance of the AI-Assisted System

As shown in Table 2, the overall accuracy of the Buffer Net for identifying a bacterial genus was 94.9%, which was the best and higher than 3D-ResNet (92.3%) [28]. The 1D-CNN was the worst, with an accuracy of only 82.6%. Meanwhile, the accuracy for 2D-CNN was moderate, whose accuracy was 91.3%. Without buffering layers, Buffer Net got a low accuracy, 88.4%. All of these results were acquired on the test dataset with over 40,000 hyperspectral images.

### 3.3. The Differentiation Speed of Our AI-Assisted HMI System

As for the speed, it took only 30 s for our HMI system to differentiate genera on microscopic hyperspectral images at the cellular level.

## 4. Discussion

An AI-assisted pathogen detection system, with designed HMI and Buffer Net, was developed for rapid, accurate and low-price automatic identification in this paper. Before us, Seo et al. and Rui et al. used HMI to classify food-borne bacteria [17,18,19,20]. Nevertheless, our method was better than the previous studies in terms of data or algorithm.

In terms of data, the wavelength range of our hyperspectral data is 440–1023 nm, and the spectral resolution is 2.1 nm, with a total of 277 bands. In contrast, the wavelength range in these researches is 450–800 nm, with a spectral resolution of 4 nm and a total of 89 bands. In addition, the maximum number of images in those studies was no more than 5000, far less than the number (>130,000) we collected. Concerning algorithms, machine learning, 1D-CNN, 2D-CNN and LSTM were utilized to extract features that would disconnect the strong spatial–spectral correlation in datacubes. Here, we proposed a new method based on 3D-CNN, called Buffer Net, to train the model directly from datacubes.

**Figure 5:**
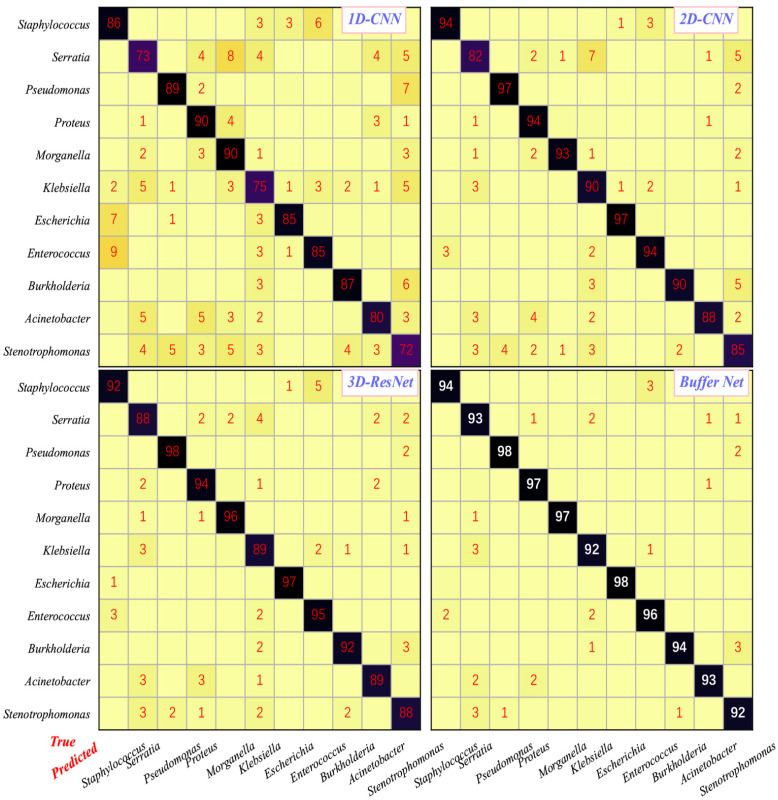
The confusion matrix of differentiating genera of Buffer Net, 1D-CNN, 2D-CNN and 3D-ResNet. The x-axis represents the genera predicted by our system, while the y-axis represents the underlying facts. Entries (*i*, *j*) indicate the percentage of samples that actually belong to class *i* that are judged to be of class *j*; entries along the diagonal indicate the discrimination accuracy of the model we developed for each genus, and off-diagonal entries indicate the misclassification accuracy. The algorithm name was presented in the top right corner of each subplot. The highest accuracy for identifying each bacterial genus was marked by bold white among these four algorithms. Accuracies with less than 1% were not presented in the confusion matrix. All classification probabilities shown in red. The probability on the diagonal represents the correct rate. In particular, white represents the highest accuracy of the four algorithms for each genus. The remaining probability that is not diagonally represents the error rate.

As for the accuracies of differentiating bacterial genera for 1D-CNN, 2D-CNN, 3D-ResNet and Buffer Net, it was no surprise that 1D-CNN has the lowest accuracy (detailed in Figure 5). In contrast, Buffer Net had the highest, because only spectral information was adopted for the 1D-CNN with spatial features discarded. Likewise, it was explainable that the accuracy of 2D-CNN was higher than that of 1D-CNN and lower than that of 3D-ResNet, because the images were considered conventional images with numerous channels, which enabled the simple usage of the spectral profiles with spatial features adopted, while 3D-ResNet could extract the features from the spatial and spectral dimensions precisely.

Noticeably, our proposed algorithm, Buffer Net, achieved the highest accuracy of 94.9%, because it was designed to learn higher-level features. It is noticeable that the differentiation precisions of Buffer Net for all genera were over 92%, with the highest precision of 98% for Pseudomonas and Escherichia. Therefore, the requirement for clinical practice was met. Buffer Net held the highest precision for identifying 11 genera compared with the others.

ResNet is a classical deep architecture that achieves a good performance in many tasks [28]. However, in our experiments, the overall accuracy of 3D-ResNet (92.3%) is 2.6% lower than Buffer Net (94.9%). Since both ResNet and Buffer Net in this paper use 3D-CNN as filters, the difference in performance can only be caused by the difference of architecture. To further demonstrate the effectiveness of the Buffer Net architecture, we practiced an ablation experiment by removing all buffering layers from Buffer Net and obtained an accuracy of 88.4% (Table 2). The accuracy of Buffer Net without the buffering layers is even less accurate than 2D-CNN (91.3%), indicating that the frequent down sampling limits the feature representation capability, which is enhanced by the buffering layers.

Compared with conventional methods in routine practice, our AI-assisted system allows for automatic and rapid bacterial identification. Since it identifies the genus of in dividual bacteria, it does not require any culture of the pathogen, as long as the individual bacteria can be isolated. In scenarios where bacterial isolation techniques are unavailable, the system can significantly reduce the cultivating time of the bacteria. Compared to expensive PCR and MALDI TOF, our method can obtain high precision while keeping the costs under control, even lower than the morphological examination. In addition, since the operation is simple and the system automates the identification, there is no expertise required for the operators. Therefore, this can reduce the cost of the clinical diagnosis for training physicians and their redundant workloads.

Recently, time-lapse coherent imaging [29], three-dimensional quantitative phase imaging [30] and holographic microscopy [31] have been used for bacterium identification. Nevertheless, time-lapse coherent imaging based on colonies is time-consuming. Three-dimensional quantitative phase imaging or the holographic mi-croscopy identifies pathogens with a refractive index (RI). HMI uses spectral and morpho-logical information to classify pathogen genera.

This study also had some limitations. Firstly, the precision of those algorithms mentioned above for identifying these four genera (Serratia, Klebsiella, Acinetobacter and Stenotrophomonas) was relatively low, and this may be due to the similarities between the four genera. Further improvements will be implemented to optimize the precisions. In addition, we focused on bacterial genera identification at the cellular level in this study, which could be further subdivided into species and subspecies. Species or subspecies identification based on datacubes of pathogens will be explored in subsequent studies, and these are what we are doing. Lastly, although over 130,000 images with 11 genera were adopted to develop the algorithm Buffer Net, far more than 11 genera need to be identified in routine clinical practice. Therefore, the number and diversity of the training and test data need to be augmented further.

## 5. Conclusions

In this study, we developed an AI-assisted system to automatically identify bacterial genera by combining the designed HMI system and Buffer Net. Utilizing the HMI system, a large-scale dataset where bacteria datacubes had a wider wavelength range and a finer spectral resolution was built to train Buffer Net for identifying 11 genera. The 3D-CNN and the architecture with buffering layers and down sampling layers contributed to the highest accuracy (94.9%) of Buffer Net compared with the other algorithms. The AI-assisted system has great potential in rapidly identifying bacteria, because it can shorten or even eliminate the cultivation time. After obtaining the microscopic datacube of an individual bacterium, it only takes 30 s to classify a pathogen.

## Figures and Tables

**Table 1:** The number of bacteria.

Genera	Numbers for Training	Numbers for Testing	Total
Stenotrophomonas	9070	3888	12,958
Escherichia	7135	3058	10,193
Morganella	7384	3165	10,549
Burkholderia	6337	2717	9054
Serratia	8126	3483	11,609
Pseudomonas	8954	3838	12,792
Acinetobacter	7185	3080	10,265
Klebsiella	11,255	4825	16,080
Proteus	11,435	4902	16,337
Staphylococcus	8182	3508	11,690
Enterococcus	8887	3810	12,697
**total**	93,950	40,274	134,224

**Table 2:** The comparison of the differentiation accuracy for all samples.

Algorithm	1D-CNN	2D-CNN	3D-ResNet	Buffer Net (Without)	Buffer Net (With)
**Accuracy**	82.6	91.3	92.3	**88.4**	**94.9**

**Red** represents the highest performance. **With/without** means Buffer Net with/without buffering layers.

## Data Availability

The data will be made available by the corresponding author upon reasonable request. The code is at https://github.com/chengltao/Buffer-Net; The trained weights is at https://drive.google.com/file/d/123AYGNOAIKJTWLv6ugSbEZ4RwSK8nN3f/view?usp=sharing.
